# Thermodynamic Stability of Histone H3 Is a Necessary but not Sufficient Driving Force for its Evolutionary Conservation

**DOI:** 10.1371/journal.pcbi.1001042

**Published:** 2011-01-06

**Authors:** Srinivas Ramachandran, Lisa Vogel, Brian D. Strahl, Nikolay V. Dokholyan

**Affiliations:** 1 Department of Biochemistry and Biophysics, University of North Carolina at Chapel Hill, Chapel Hill, North Carolina, United States of America; 2 Program in Molecular and Cellular Biophysics, University of North Carolina at Chapel Hill, Chapel Hill, North Carolina, United States of America; Harvard University, United States of America

## Abstract

Determining the forces that conserve amino acid positions in proteins across species is a fundamental pursuit of molecular evolution. Evolutionary conservation is driven by either a protein's function or its thermodynamic stability. Highly conserved histone proteins offer a platform to evaluate these driving forces. While the conservation of histone H3 and H4 “tail” domains and surface residues are driven by functional importance, the driving force behind the conservation of buried histone residues has not been examined. Using a computational approach, we determined the thermodynamically preferred amino acids at each buried position in H3 and H4. In agreement with what is normally observed in proteins, we find a significant correlation between thermodynamic stability and evolutionary conservation in the buried residues in H4. In striking contrast, we find that thermodynamic stability of buried H3 residues does not correlate with evolutionary conservation. Given that these H3 residues are not post-translationally modified and only regulate H3-H3 and H3-H4 stabilizing interactions, our data imply an unknown function responsible for driving conservation of these buried H3 residues.

## Author Summary

Most proteins fold to a well-defined, three-dimensional structure, which can be delineated into the protein surface and its buried core. When comparing amino acid sequences of the same protein from different organisms, we would expect to find certain residue positions conserved due to the importance of that position in either maintaining the protein's function or its three-dimensional structure. In this study, we looked at residues in the buried core domains of histone proteins H3 and H4, which have no known function other than maintaining the three-dimensional structure of the protein. We find that perturbing protein stability (which is a measure of maintenance of the protein's structure) by mutating these residues compromises survival fitness in yeast. However, the stability conferred by buried amino acids of H3 alone cannot account for their evolutionary conservation, which is in striking contrast to other proteins where stability has been shown to be the driving force for sequence conservation. This conservation of H3 thus points to either new additional functions of H3 that have not been uncovered or a unique conservation mechanism that goes beyond survival pressure. These data therefore reveal a highly conserved domain that is distinct in its evolutionary conservation.

## Introduction

In eukaryotes, histone and non-histone proteins package genomic DNA into higher order chromatin structures. These higher order structures control the accessibility of genomic DNA to various cellular machineries that perform transcription, replication, repair and recombination. The fundamental unit of eukaryotic chromatin is the nucleosome, composed of ∼147 base pairs of DNA wrapped around the histone octamer [1]. The histone octamer comprises of two copies of each of the four histone proteins: H2A, H2B, H3, and H4. All these histone proteins are characterized by the “histone fold”, consisting of the alpha-helical and globular “handshake” motif in between the unstructured N- and C-terminal “tails” [2]. The “handshake” motif helps in stable dimerization of H2A-H2B and H3-H4. (H3-H4)_2_ forms a stable tetramer, where two H3-H4 dimers are arranged symmetrically across an interface formed by adjacent H3 molecules (H3,H3′; Figure S1A, B). Similarly, the H2A-H2B dimer contacts the H3-H4 dimer through a beta-strand extension between H2A and H4, which serves as the only region of contact between these dimers (Figure S1C, D). Thus, the buried region of the histone octamer can be identified as either the buried residues of the dimers, or the residues that form the interfaces between the dimers while forming the tetramer and octamer.

The amino acid sequences of histone proteins are highly conserved from yeast to humans: H3 and H4 feature more than 90% conservation across all their known sequences. Covalent modifications to the histone “tail” domains [3], which regulate chromatin organization and function, drive their high sequence conservation, while the need to maintain interactions with genomic DNA may drive the sequence conservation of many residues on the surface of the histone octamer. Due to the lack of any other function, we could hypothesize that the conservation of buried and inter-histone interface residues (see Figure S1 and Figure S2) is driven by the need to maintain inter-histone interactions and to preserve the histone fold [4], [5], [6], [7], [8]. In this study, we test this hypothesis by exploring the correlation between a residue's evolutionary conservation and its contribution to the thermodynamic stability of the histone octamer.

## Results

### Thermodynamic destabilization of the histone octamer correlates with lethal phenotypes in yeast

To test the hypothesis that thermodynamic stability drives evolutionary conservation of buried and interface residues in H3 and H4, we calculated the energetic consequences of mutating each of these residues. The contribution of the H3 and H4 interface residues (see Figure S1) to stability was determined by calculating the change in stability (ΔΔG) after mutating each of the histone interface residues in H3 (H113, A114, L126, A127, I130, and R131) and H4 (T96, L97, Y98, and G99 in H4) to all possible amino acids using Medusa [8], [9], a computational protein design toolkit. We then used the ΔΔG values to determine the propensity of each possible amino acid to be stabilizing at each of these positions. The amino acid with the lowest ΔΔG at a position has the highest propensity to be stabilizing at that position. We find that most of the residues present in the H3-H3′ interface (residues 113, 126, 127 and 130) have a strong preference for the native amino acid or for conserved substitutions.

**Figure 1:**
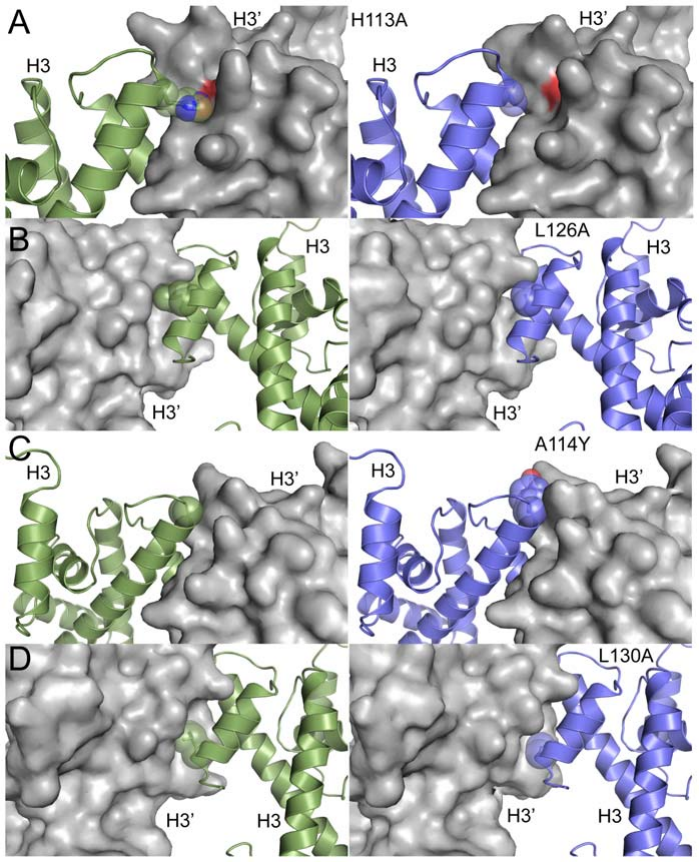
Inter-histone H3-H3′ interface is destabilized by mutagenesis. Models for H3-H3′ interface mutations that were tested in this study (right panels) are shown in comparison to the wild type interface (left panels). One of the binding partners is shown in surface representation, while the other is shown in cartoon representation with spheres depicting the mutated residues. H113A (**A**) results in the loss of a hydrogen bond with D123 across the interface, L126A (**B**), results in loss of hydrophobic interactions across the interface, A114Y (**C**) introduces bulky side-chain in the interface, and L130A (**D**) also results in loss of hydrophobic contacts across the interface. The structures were rendered using PyMOL (http://www.pymol.org).

**Figure 2:**
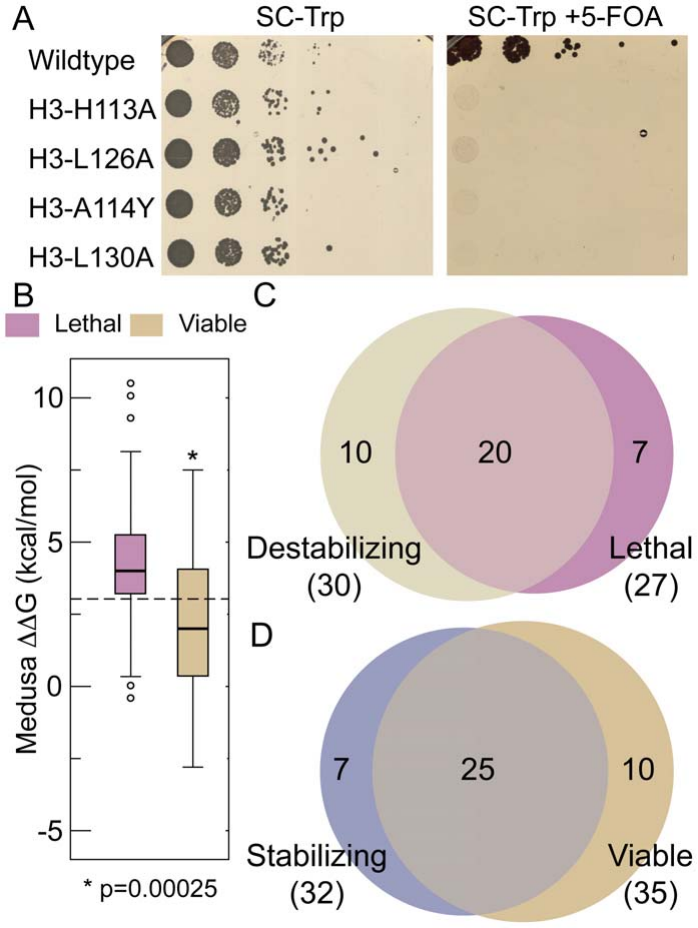
Thermodynamic destabilization of the histone octamer correlates with lethal phenotypes in yeast. **A**, Yeast strains bearing H3-H3′ interface mutations predicted to cause thermodynamic destabilization of the nucleosome are lethal. The WZY42 histone shuffle strain transformed with the wild-type or indicated H3 mutant was plated in 10-fold serial dilution on selective synthetic complete-Trp media with (right plate) or without (left plate) 5-Fluoroorotic acid (5-FOA). **B**, Broad analysis of growth phenotypes and their relation to predicted nucleosome stability reveal significant difference in ΔΔG between viable and lethal mutants. Box plots are shown, which represent range between 25 and 75 percentile values. Horizontal line inside the box represents the median. Whiskers correspond to values nearest to 1.5 times the interquartile range and outliers are represented as circles. P-value is obtained from two sample, single-tailed t-test. The dashed-line represents ΔΔG of 3kcal/mol, used to distinguish between stabilizing and destabilizing mutants. **C**, Venn diagram showing the significant overlap that exists between lethal and destabilizing mutants found in H3 and H4 for interface and buried residues. Compilation of lethal mutant results is from HistoneHits database. The numbers inside the Venn diagram refer to number of mutations belonging to the corresponding categories. **D**, Venn diagram showing the significant overlap that exists between viable and stabilizing mutants found in H3 and H4 for interface and buried residues. Compilation of lethal mutant results is from HistoneHits database. The numbers inside the Venn diagram refer to number of mutations belonging to the corresponding categories.

**Figure 3:**
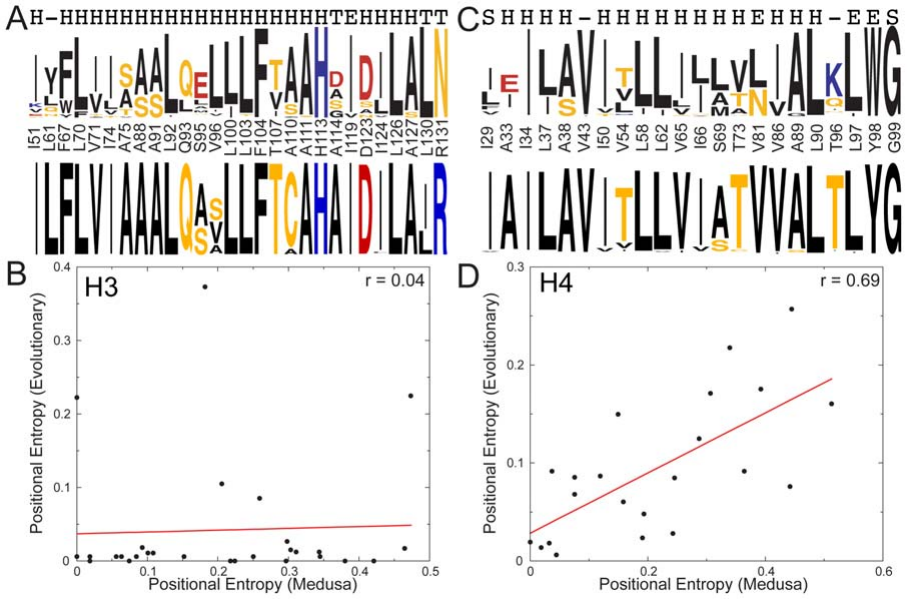
Evolutionary and calculated sequence entropies have significant correlation in H4 but no correlation in H3. **A**, **C**, Sequence logo (http://weblogo.berkeley.edu/) of propensities of different amino acids calculated using Medusa at each of the buried/interface residues show that Medusa recapitulates 75% of positions in H3 (**A**, top logo) and 54.5% of positions in H4 (**C**, top logo). For comparison, the corresponding sequence logo from evolutionary conservation (obtained from homology-derived secondary structure of proteins (HSSP) database) is also shown for H3 (**A**, bottom logo) and H4 (**C**, bottom logo). The amino acids are colored according to their physical property (hydrophobic amino acids are colored black, negatively charged red and so on). The secondary structure corresponding to each amino acid is shown at the top: helix (H), beta strand (E), hydrogen bonded turn (T), bend (S) or no secondary structure (-). **B**, **D**, Plotting positional entropy of buried residues in H3 and H4 calculated using Medusa against evolutionary positional entropy shows no correlation for H3 (**B**) and significant correlation for H4 (**D**). Each point in the plots represents a specific buried/interface residue. The actual values of Evolutionary and Medusa entropies are represented in Supplementary Tables 3 and 4.

Since evolutionary pressure is associated with survival fitness, we asked if engineered mutations that should result in thermodynamic destabilization of the histone octamer would lead to phenotypic consequences in the budding yeast *Saccharomyces cerevisiae*. We made three H3-H3′ interface mutations (Table 1) that disrupted conserved interactions (*H113A*, *L126A* and *L130A*) and one interface mutation that introduced a non-preferred amino acid at that position (*A114Y*). Based on our models (Figure 1A, where the left panels represent WT interface and right panels represent the mutant interface), *H113A* disrupted hydrogen-bonding and hydrophobic interactions of H113 with a negatively charged pocket formed by the adjacent H3 surface. *L126A* and *L130A* each disrupted hydrophobic interactions with the adjacent H3 (Figure 1B, D). Consistent with these mutations resulting in a significant destabilization of the H3-H3′ interactions *in silico* (Table 1), we found that these H3 mutations were lethal when engineered in budding yeast (Figure 2A). Regarding H3A114, our calculations predict the preference of small amino acids or aspartate at this position (Figure 3A), as large amino acids at 114 would disrupt H3-H3′ interaction (Figure 1C). Consistent with this idea, a *A114Y* mutation was also lethal in yeast (Figure 2A). The interface residues in H4 on the other hand interact with V101-A104 of H2A primarily through backbone hydrogen bonds (Figure S1C, D). The side chain of H4Y98 is embedded in a deep groove formed on the H2A surface, which makes the position suitable only for aromatic residues as revealed by our calculations. In contrast, H4G99 is placed in a position where any side chain would have significant steric clashes with the H2A surface, making the position amenable only to glycine (Figure 3C). We thus find that the selected interface residues lining the H3-H3′ and the H4-H2A interfaces are important for nucleosome stability *in silico* and viability *in vivo*.

**Table 1:** List of mutations in H3 screened in this study.

Mutation	Medusa ΔΔG (kcal/mol) ± SEM^1^	Predicted Phenotype	Experimental Result
H3 H113A	8.1±0.5	Lethal	Lethal
H3 L126A	4.8±0.4	Lethal	Lethal
H3 A114Y	3.0±0.4	Lethal	Lethal
H3 L130A	10.1±0.4	Lethal	Lethal

1 The standard error of mean (SEM) obtained for the Medusa calculations.

To extend this comparison between residues needed for nucleosome stability and their effects on growth in yeast, we calculated the change in nucleosome stability of 62 mutations pertaining to buried/interface residues extracted from the HistoneHits database [10]. Overall, we observed that residues found to be essential for viability are often also associated with being required for nucleosome stability (Figure 2B, Table S1 and Table S2). The ΔΔG of lethal mutants is significantly higher than the ΔΔG of viable mutants: the probability that the ΔΔG of lethal and viable mutants are similar is 2.5×10^−4^, indicating that the difference in destabilizition of lethal and viable mutants is statistically significant. These results imply that the lethality observed in these studies is due to thermodynamic destabilization of the nucleosome. Furthermore, if we use an arbitrary ΔΔG cut-off of +3 kcal/mol as a threshold to suggest a point where sufficient thermodynamic destabilization would lead to lethality, we are able to predict 74% of the lethal mutants and 71% of the viable mutants from the HistoneHits database (Figure 2C, D). These data suggest that nucleosome stability, which is essential for viability, is a major driving force behind the sequence conservation of buried H3 and H4 residues.

### Evolutionary and calculated sequence entropies have significant correlation in H4 but no correlation in H3

To explore the relationship between thermodynamic stability and sequence conservation of buried and interface histone residues, we compared the sequence entropies [4] (see Methods for mathematical definition) of these residues from our calculations to those observed in evolution. We use sequence entropy as a parameter to measure the degree of variability of amino acids at a given position across a wide range of species. Highest sequence entropy would indicate all twenty amino acids are equally probable in that position, while zero value entropy would indicate complete conservation of that position. We calculated the sequence entropy at each buried and interface position in H3 and H4 based on the ΔΔG of all possible amino acids at that position (detailed in Methods), which we compared to the evolutionary sequence entropy obtained from the homology-derived secondary structure of proteins database [11]. There could be a possible bias for native amino-acid type in our calculations because we keep the backbone fixed. However, since the histone-fold is highly conserved (the Cα root mean square deviation (RMSD) between the crystal structures of yeast [12], drosophila [13], xenopus [1] and human [14] nucleosomes range between 0.25–0.5 Å, indicating very high structural similarity), we expect this bias to be minimal.

We found statistically significant correlation between Medusa and evolutionary entropy for buried and interface residues in histone H4 (Figure 3D, r = 0.69, p = 3.8×10^−4^, Table S4), while there is no correlation in the case of histone H3 (Figure 3B, Table S3). We observe that thermodynamically, native residues in H3 are preferred in 75% of the positions considered (Figure 3A), as compared to 54.5% for H4 (Figure 3C). Compared to 63.4% [8] and 57.1% [15] of native residues preferred in the buried region of other proteins, H3 has a highly optimized buried-core. However, even such high sequence recapitulation is not accompanied by correlation between Medusa sequence entropy and evolutionary entropy of H3 (Figure 3B).

To analyze the lack of correlation in H3 further, we divide the H3 residues that we consider into three sets (Figure S3). The first set corresponds to five positions that feature much higher evolutionary entropy compared to other positions (Figure S3A). These five outliers are S95, V96, A110, I124 and L130. Evolutionarily, cysteine is the most conserved amino acid at position 110, but we do not consider cysteine in our calculations, hence we do not analyze this position further. To explore functional constraints on the evolution of S95, V96, I124 and L130, we analyzed their tree-based conservation. We observe that the conservation of S95, V96 and L130 (Figures S4, Figure S5 and Figure S6 respectively; the organisms whose sequences were used to construct the tree are shown in Figure S7) is highly tree-determinant, implying increasing the stability of the nucleosome to not be a major driving force. The observed tree-dependent evolution implies a functional constraint for conservation in these positions. In the second set, we observe twelve positions whose stability seemingly correlates with evolutionary conservation (Figure S3B, r = 0.64, p = 0.025). However, the low value of the slope of the linear fit (0.032) indicates that evolutionary conservation is higher than the conservation expected due to stability, even if it follows the same trend as stability. The third set corresponds to eleven positions that feature evolutionary conservation that is much higher than required for stability. Nine of these positions are buried (Table S5), while two belong to the interface. Many buried positions being conserved much more than required by stability indicates that thermodynamic stability is not a sufficient driving force for H3 conservation.

### Spatially remote and proximal pairs of residues coevolve in H3

**Figure 4:**
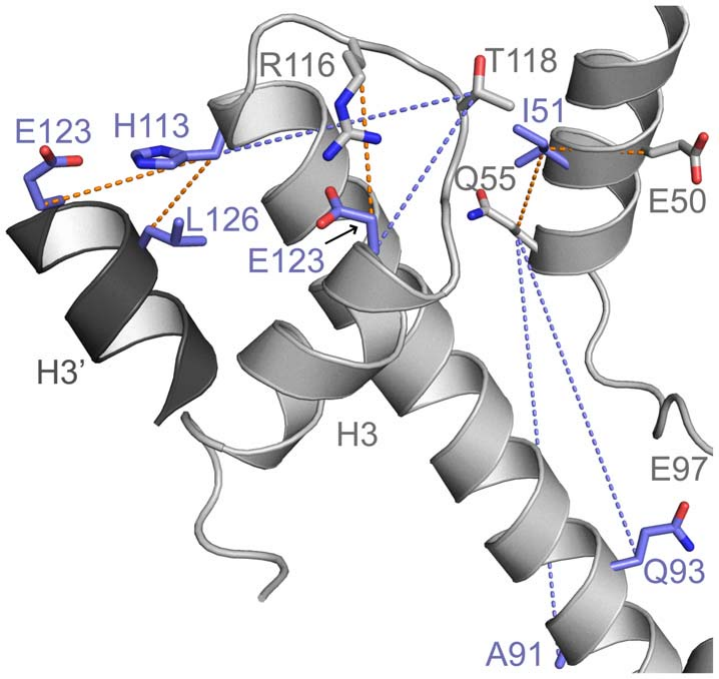
Coevolution of spatially remote and proximal pairs of residues in H3 suggest a function of the buried H3 residues independent of mediating stability. Significantly coevolving pairs of residues are shown in stick representation with dotted line between their Cβ atoms. Buried/interface residues are colored blue, while the rest of the protein is colored in grey and shown using the cartoon representation. The dotted lines between residues that are spatially proximal are colored orange, while the dotted lines between spatially distant residues are colored blue. The structure was rendered using PyMOL (http://www.pymol.org).

The increased sequence conservation observed in the buried residues of H3 is only observed in other proteins when those residues are essential for a protein's function other than stability [8]. An indirect way to assess if these residues are implicated in function is through evolutionary analysis that picks coevolving residues. Coevolution of two residues that are spatially distant may point to evolutionary constraints due to functional roles [16]. To find such coevolving residues in H3, we used multiple sequence alignment of H3 sequences from a wide range of species (from yeast to human, 223 species in total) to calculate the Z-score of normalized mutual information of any two positions in H3. A Z-score higher than 4 indicates significant coevolution [17]. In H3, we find that there are only ten pairs of coevolving residues with significant Z-scores. Of these ten pairs, nine pairs (Table 2) have at least one buried/interface residue that we consider in this study and out of these nine pairs, five pairs form structural contacts either intramolecularly or across the H3-H3′ dimeric interface (Figure 4). Notable among these pairs of residues that are spatially proximal are the electrostatic interaction between H113 and E123 and the hydrophobic interaction between H113 and L126, both of which occur across the dimeric H3-H3′ interface. Additionally, R116 and E123, which form an inter-molecular salt-bridge are also found to coevolve. The disruption of salt-bridge between R116 and E123 by the mutation R116H has been identified as a sin mutant, which alleviates the requirement for nucleosome-remodeling factors in transcription activation [18], [19]. Thus, our coevolution analysis identifies at least one functionally important coupling. The coevolving pairs of residues that do not form structural contacts could have coevolved due to i) their involvement in the folding kinetics of H3, ii) due to negative design [20], where control of interactions between these residues is required for elimination of non-native structures, or iii) due to other functions that involve the residues in the identified pairs, implying a functional constraint on their evolutionary conservation.

**Table 2:** List of residues coevolving in H3.

Residue Pair^1^	Z-Score^2^	P-value^3^
Spatially Proximal		
E50-I51	4.2	0.029
I51-Q55	7.9	0.041
H113 -D123	4.0	0.048
H113-L126	4.0	0.029
R116-D123	4.6	0.042
Spatially Distant		
T118 -D123	7.2	0.026
Q-55, A-91	5.2	0.022
Q-55, Q-93	7.0	0.046
H-113, T-118	4.5	0.022

1 Coevolving pairs of residues.

2 Z-scores of normalized mutual information between any two residues. Calculation of Z-scores is described in Methods.

3 P-value denotes the probability of obtaining normalized MI upon randomly shuffling one of the positions (detailed in Methods).

## Discussion

Our data point to a force apart from thermodynamic stability that conserves buried and interface residues in H3. This driving force of conservation found in buried H3 residues has not been observed or characterized in buried residues of other highly conserved proteins to our knowledge. Statistically significant correlation between Medusa derived sequence entropies and evolutionary entropies has been shown before in other proteins [8], and in H4 in this study, implying stability as a driving force for conservation buried residues. Further, when we performed similar analysis on all buried positions of actin (PDB ID 1J6Z) and tubulin (PDB ID 1Z5V), we observe statistically significant correlation between evolutionary and Medusa entropies (Table 3). Thus, there is correlation between thermodynamic stability and evolutionary conservation in the buried residues found in other highly conserved proteins, namely actin and tubulin. These results reveal H3 as the only protein known so far, whose conservation of core residues is not driven by stability alone. We therefore suggest there is a novel function associated with the buried and interface residues of H3 that is driving the unique level of conservation.

**Table 3:** Correlation between evolutionary entropy and Medusa entropy in highly conserved proteins.

Protein	Average Evolutionary Entropy^1^	Correlation Coefficient^2^	P-Value of correlation co-efficient^3^
Histone H3	0.04	0.04	0.84
Actin	0.07	0.36	0.009
Histone H4	0.09	0.69	3.80×10^−4^
Tubulin	0.16	0.37	3.85×10^−4^

1 Average over all buried residues in the protein to indicate extent of conservation of the buried core – lower value indicates higher conservation.

2 Pearson correlation coefficient when comparing Medusa derived entropies and Evolutionary entropies obtained from the HSSP database.

3 P-value testing the null hypothesis that the correlation between Medusa derived entropies and evolutionary entropies is due to pure chance.

What might be the function of the conserved buried residues in H3? Given these residues are not found to be post-translationally modified, and their strict conservation is independent of H3-H3′ and H3-H4 stability, it could be suggested that they may be playing a role in histone chaperone interactions and deposition. Asf1 is one such histone chaperone that facilitates the deposition of histones in chromatin during replication. Even though Asf1 binds and/or competes for the H3-H4 dimer by interacting with residues in the H3-H3′ interface [21], it cannot account for the conservation of the residues it interacts with in H3, as the interacting residues from Asf1 are not similarly conserved. In addition, Asf1 is not known to interact with the H3 buried residues we examined. We do not rule out the possibility that other histone chaperones or other histone interacting proteins interact with the buried and interface residues in H3/H4 for a functional purpose, but it is striking to note that even though most of these proteins may also interact with H4, conservation of H4 can be accounted for by thermodynamic stability alone.

Another possibility for the increased conservation of buried residues of H3 could be the need to tightly regulate and fine-tune the stability of nucleosomes during transcriptional regulation, as a slight increase or decrease of nucleosome stability could have profound effects on cellular processes like transcription. We find evidence for this hypothesis in the observation that H3 variants such as H3.3 function by modulating nucleosome stability [22]. Although the core of H3.3 differs from canonical H3 in humans at just three positions, the destabilization of H3.3 containing nucleosomes has been shown to be important in transcriptional regulation [22]. Thus, major changes in cellular function due to minor perturbations in H3 sequence suggest the need for tight control of nucleosome stability. Such control may explain why the core residues of H3 are so highly conserved.

We conclude that an unknown set of factors is driving conservation of H3 to a degree that has not been found in any other protein to date. The significance of these residues outside of histone fold interactions awaits further discovery. Our finding of an unexpected level of sequence conservation, not demonstrated before in a protein to our knowledge, suggests the ability to predict functional roles of amino acid residues apart from imparting thermodynamic stability to a given protein.

## Materials and Methods

### Choice of histone residues examined

The residues analyzed in this paper include 51, 61, 67, 70, 71, 74, 75, 88, 91, 92, 93, 95, 96, 100, 103, 104, 107, 110, 111, 113, 114, 119, 123, 124, 126, 127, 130 and 131 from H3 and 29, 33, 34, 37, 38, 43, 50, 54, 58, 62, 65, 66, 69, 73, 81, 86, 89, 90, 96, 97, 98 and 99 from H4. The interface residues include 110, 113, 114, 123, 126, 127, 130 and 131 in H3 and 96, 97, 98 and 99 in H4. We performed ΔΔG calculations on two types of residues in H3 and H4, i) residues in the interface of H3-H3′ and H4-H2A and ii) residues that are buried in the histone octamer. We define buried residues as those that have less than 1 Å^3^ exposed solvent accessible surface area in the histone octamer. We calculate solvent accessible surface area using the method of LeGrand and Merz [23], using 1024 dots on surface of each atom. We define interface residues (lining H3-H3′ and H4-H2A) as those that formed persistent contacts across these interfaces in our earlier simulations of the mono-nucleosome [24].

### ΔΔG Calculations using Medusa

We used the coordinates of histone octamer, extracted from the crystal structure of the yeast nucleosome [12] (PDB ID 1id3) to perform Medusa calculations. Since we consider only core residues of H3 and H4 that do not interact with DNA, we do not consider DNA in our calculations. Medusa calculations involve a Monte-Carlo based simulated annealing procedure that uses rotamer libraries of amino acids for fast minimization of its energy function while leaving the backbone fixed. For all the residue positions we considered in this study, ΔΔG was calculated for mutation of the native amino acids at that position to 17 other amino acids (all natural amino acids except cysteines and prolines: we do not consider disulfide bonds in our model and prolines can also affect the protein backbone, which we hold fixed in our calculations). For each position, residues within 10 Å (CA – CA distance) were allowed to sample all available native rotamers, while rest of the residues were allowed to sample the sub-rotameric states of the starting rotamer. We averaged the free energy obtained from 100 Medusa calculations to obtain ΔΔG for each mutation. We define ΔΔG as:
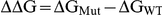
Where ΔG_Mut_ is the stability of the mutant and ΔG_WT_ is the stability of the wild type. Thus, a destabilizing mutation would result in a positive ΔΔG. For buried residues, we calculated only the ΔΔG for each mutation. For the interface residues, we calculate the difference in binding energy between mutant nucleosome and the wild-type nucleosome; the difference in binding energy is positive if mutation results in a decrease in binding energy. For comparison between ΔΔG and viability, we calculated the change in nucleosome stability of 62 mutations pertaining to buried/interface residues extracted from the HistoneHits database [10]. The difference in between lethal and viable mutants were statistically tested using a two sample, one-tailed t-test (sample size of lethal mutants = 27 and viable mutants = 35) which gives a p-value of 2.5×10^−4^.

### Sequence entropy from ΔΔG

We assume a Boltzmann distribution of amino acid residues in a given position, where the ratio of propensity of two amino acids can be calculated from the ΔΔG of mutating one of the amino acids to the other:
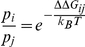
where p_i_ and p_j_ are the propensities of amino acids i and j at a given position and ΔΔG_ij_ is the free energy change upon mutating i to j at that position obtained from Medusa calculations. Here, the temperature T refers to the physical temperature at which the protein exists and functions in the organism, and hence would vary in a narrow range. We use a temperature of 300 K to perform all calculations. These propensities are in turn used to calculate the sequence entropy at a given position:
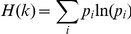
where H(k) is the sequence entropy at position k and p_i_ is the propensity of amino acid i at position k. The positional sequence entropy thus calculated is compared to evolutionary entropy extracted from homology-derived secondary structure of proteins (HSSP) database entry for yeast nucleosome (1id3.hssp) [11].

### Mutual information

We use mutual information as a measure of coevolution of two positions in either the same protein or across two proteins [25]. Mutual information is defined as:
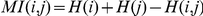
where MI(i,j) is the mutual information of positions i, j; H(i) is the sequence entropy at position i and H(i,j) is the joint entropy of positions i and j. Since MI by definition cannot be greater than Min(H(i),H(j)), it correlates with the individual and joint entropies. To remove dependence of MI(i,j) on H(i) or H(j), we normalize MI(i,j) by dividing it by H(i,j) to compare positions with varying entropies. It was shown before that H(i,j) was the best normalizing factor compared to H(i) and H(j) [17]. We then calculate the Z-score for the normalized MI for a given protein or protein pair. Z-score is defined as:
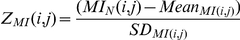
In the above equation, MI_N_(i,j) refers to normalized MI. To account for evolutionary noise in MI_N_(i,j), we perform tree based shuffling of position j, while keeping position i constant as described by Noivirt *et al.* [26]. The probability of shuffling position j between sequence a and sequence b is given by:
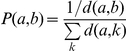
where d(a,b) is the genetic distance between sequence a and b and is obtained from Clustalw2 [27]. Clustalw2 calculates genetic distance based on the minimum number of substitutions required to convert one sequence into another (with correction applied to allow multiple substitutions to have occurred [28]). We perform 100 iterations of 2000 shuffles for each pair of positions for which we observe significant Z-score. We construct a distribution of MI_N_(i,j) from these shuffles and then determine the probability of obtaining the evolutionarily observed MI_N_(i,j) from the distribution of MI_N_(i,j) determined through shuffles (P-value). A P-value of less than 0.05 is considered statistically significant.

### Sequence acquisition

Swissprot IDs for Histones H3 sequences from different species were obtained from its Interpro family (Interpro accession ID: IPR000164; website: http://www.ebi.ac.uk/interpro/). The sequences were pruned to remove duplicates and fragments. We then selected a unique sequence for each species in the remaining group of sequences and performed multiple sequence alignments using Clustalw2 [27]. For the analysis of mutual information, we used sequences from 223 species for H3. We use Henikoff position-based weighting algorithm to remove bias due to phylogenetic proximity [29].

### Yeast strains, plasmids and growth assays

*S. cerevisiae* strains used in this study are summarized in Table S6. Ycp50-copy II (HHT2-HHF2), a plasmid containing wild-type H3-H4-copy II alleles with their native promoters and a TRP selectable marker was used for the mutational analyses. Point mutations were generated using site-directed mutagenesis (QuickChange II kit; Agilent Technologies). Oligonucleotide primers were designed using the wild-type *S. cerevisiae* gene sequence of H3 or H4 found in the Saccharomyces Genome Database sequence for HHT2 and HHF2. Mutant plasmids were sequenced for accuracy prior to performing histone shuffling in the yeast strains WZY42 as described by Zhang *et al.* [30] To analyze the effects of these mutants on yeast growth and viability, wild type and mutant strains were grown to an optical density of 0.5 at 600 nm in SC-Trp media prior to performing ten-fold serial dilutions on SC-Trp and 5-FOA plates. Plates were incubated at 30°C for 48–72 hours before being photographed. To confirm that our results were not strain-specific, we analyzed the same H3 mutants in YBL574 strain background (Table S5) and obtained identical results.

## Supporting Information

Figure S1 Inter-histone interfaces of H3 and H4. The crystal structure of the yeast nucleosome (PDB 1id3) is rendered in cartoon representation (a, c). The H3-H3′ interface is shown in b and the H4-H2A interface is shown in d. H3 and H3′ form a homo-dimer. The H4-H2A interface is formed by a short beta sheet, whose hydrogen bonds are denoted with dashed lines in d. The residues considered in this study are shown in stick representation and are labelled. The structures were rendered using PyMOL (http://www.pymol.org).
(0.65 MB JPG)

Figure S2 Structure of the yeast mononucleosome and location of H3 and H4 buried and interface residues. The crystal structure of the yeast nucleosome (PDB 1id3) is rendered in cartoon representation and consists of 147 base pairs of DNA wrapped around two copies of each of the four core histone proteins: H2A, H2B, H3, and H4. The buried residues of H3 and H4 are represented as scaled spheres. H3 is colored blue and H4 is colored green. The structure was rendered using PyMOL (http://www.pymol.org).
(0.29 MB JPG)

Figure S3 Distinct conservation profiles of three sets of residues in H3. Residues that feature much higher evolutionary entropy compared to other positions are shown as red circles (A). Residues featuring low evolutionary entropy, indicating conservation much higher than required by stability are indicated as blue triangles (A,B). Residues featuring evolutionary entropy that has modest correlation with Medusa-derived entropy are shown as black open squares (A,B). The red line indicates the linear regression (r = 0.64) between Medusa-derived entropy and evolutionary entropy for positions shown in black open squares (B).
(0.49 MB TIF)

Figure S4 Tree-based conservation profile of position 95 in H3. The conservation of position 95 is determined at each node of the phylogenetic tree constructed from the multiple sequence alignment of H3. We observe that the nodes representing species mostly from kingdom Fungi, have a different preferred amino acid (Serine) compared to other nodes (Alanine), indicating tree-based inheritance.
(1.52 MB TIF)

Figure S5 Tree-based conservation profile of position 96 in H3. The conservation of position 96 is determined at each node of the phylogenetic tree constructed from the multiple sequence alignment of H3. We observe that the nodes representing species mostly from kingdom Fungi, have a different preferred amino acid (Serine) compared to the node containing plant kingdom (Alanine), the node containing Homo sapiens and many species of genus Drosophila (Serine), indicating tree-based inheritance.
(1.54 MB TIF)

Figure S6 Tree-based conservation profile of position 130 in H3. The conservation of position 130 is determined at each node of the phylogenetic tree constructed from the multiple sequence alignment of H3. We observe that the nodes representing species mostly from kingdom Fungi, have a different preferred amino acid (Leucine) compared to other nodes (Isoleucine), indicating tree-based inheritance.
(1.52 MB TIF)

Figure S7 Organisms whose H3 sequences were used to construct the phylogenetic tree. The color coding of organisms is based on the nodes represented in the tree presented in Figures S3,S4 and S5.
(2.83 MB TIF)

Table S1 List of mutations in H3 extracted from the HistoneHits database.
(0.05 MB DOC)

Table S2 List of mutations in H4 extracted from the HistoneHits database.
(0.05 MB DOC)

Table S3 Evolutionary and Medusa positional entropy values of buried and interface residues of H3.
(0.05 MB DOC)

Table S4 Evolutionary and Medusa positional entropy values of buried and interface residues of H4.
(0.04 MB DOC)

Table S5 List of residues in H3 that feature high conservation. 1 Entropy values obtained from HSSP database have been normalized by ln(20), the maximal possible entropy, so that the range of entropy values is between 0–1. 2 Normalized entropy obtained using the residue propensities in Medusa calculations as described in the Methods.
(0.04 MB DOC)

Table S6 Yeast strains used in the study.
(0.04 MB DOC)
