# Supplementary figures and images for: Thermodynamic Stability of Histone H3 Is a Necessary but not Sufficient Driving Force for its Evolutionary Conservation
Source: PLoS Comput Biol. 2011 Jan 6;7(1):e1001042. doi: 10.1371/journal.pcbi.1001042 (PMC3017104; doi:10.1371/journal.pcbi.1001042)

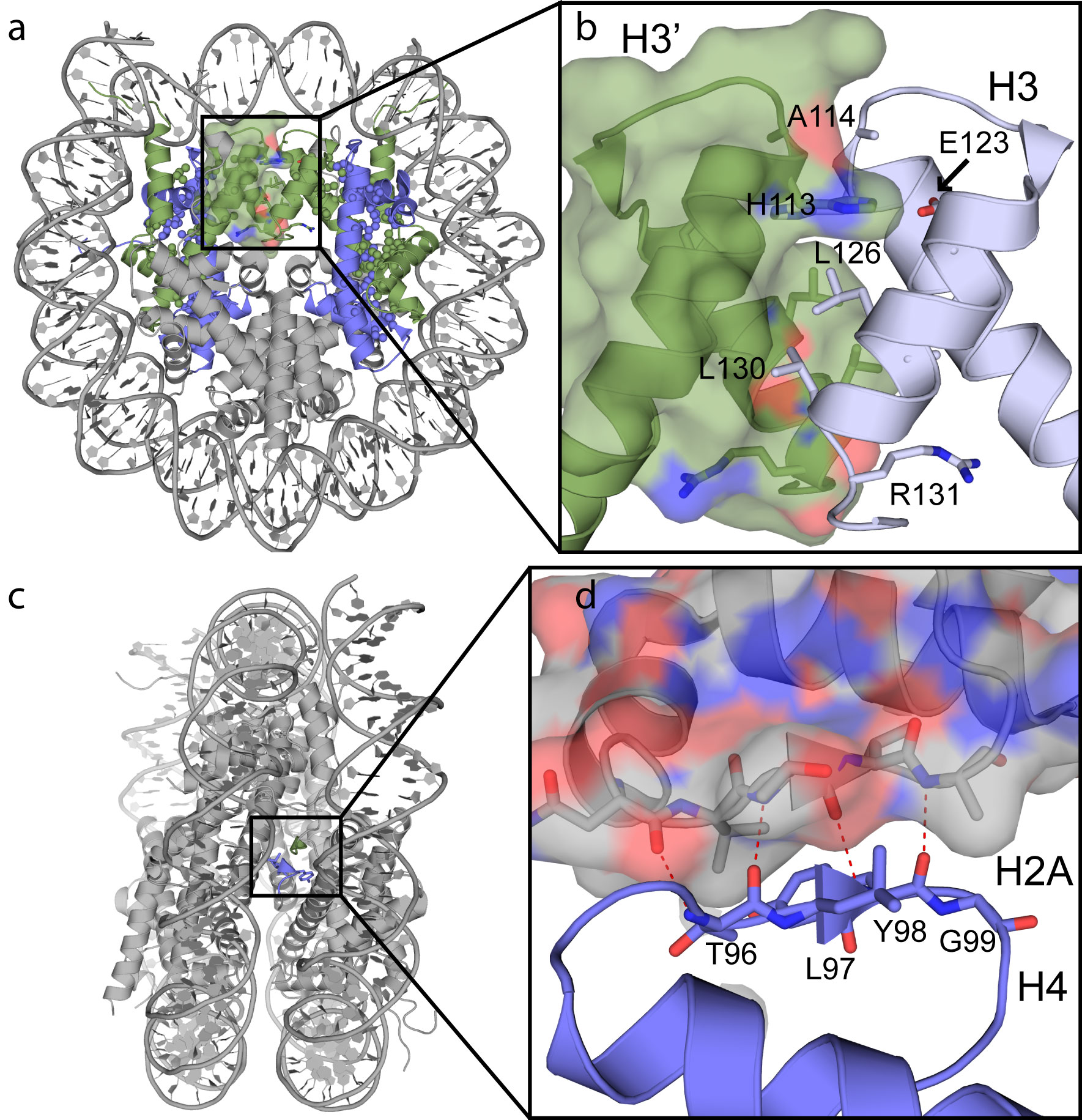

Supplement: Figure S1 — Inter-histone interfaces of H3 and H4. The crystal structure of the yeast nucleosome (PDB 1id3) is rendered in cartoon representation (a, c). The H3-H3′ interface is shown in b and the H4-H2A interface is shown in d. H3 and H3′ form a homo-dimer. The H4-H2A interface is formed by a short beta sheet, whose hydrogen bonds are denoted with dashed lines in d. The residues considered in this study are shown in stick representation and are labelled. The structures were rendered using PyMOL (http://www.pymol.org). (0.65 MB JPG) [file pcbi.1001042.s001.jpg]

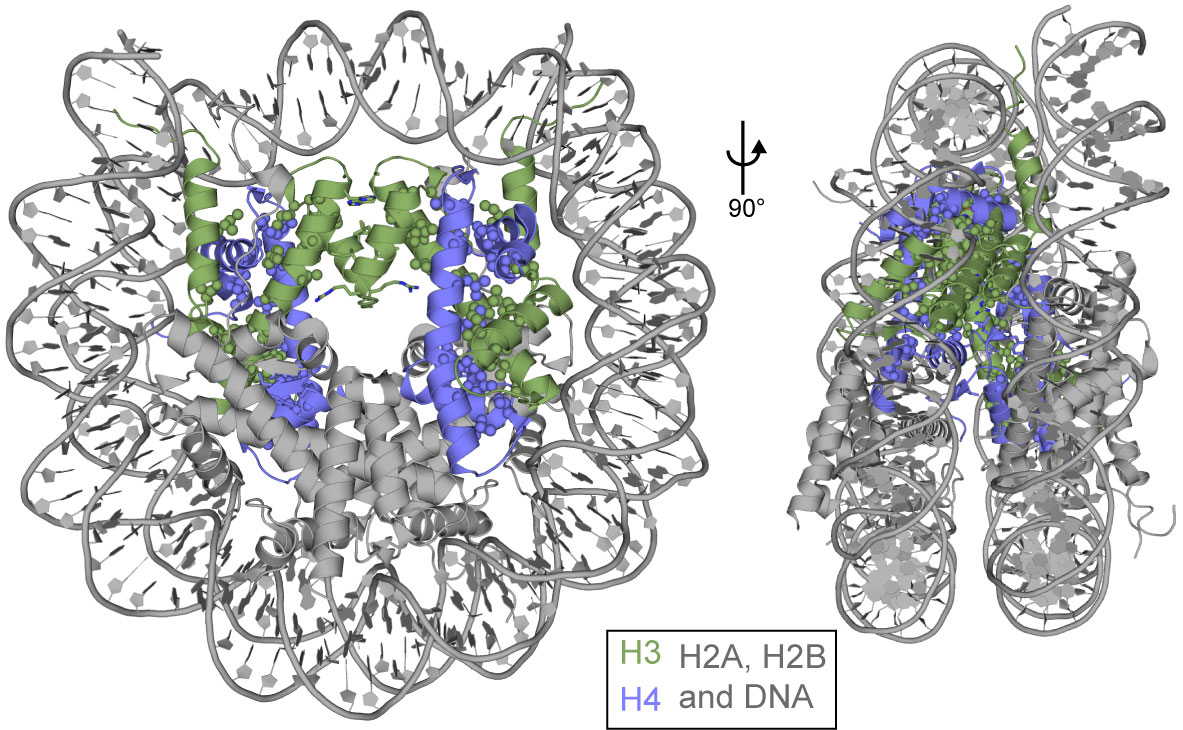

Supplement: Figure S2 — Structure of the yeast mononucleosome and location of H3 and H4 buried and interface residues. The crystal structure of the yeast nucleosome (PDB 1id3) is rendered in cartoon representation and consists of 147 base pairs of DNA wrapped around two copies of each of the four core histone proteins: H2A, H2B, H3, and H4. The buried residues of H3 and H4 are represented as scaled spheres. H3 is colored blue and H4 is colored green. The structure was rendered using PyMOL (http://www.pymol.org). (0.29 MB JPG) [file pcbi.1001042.s002.jpg]

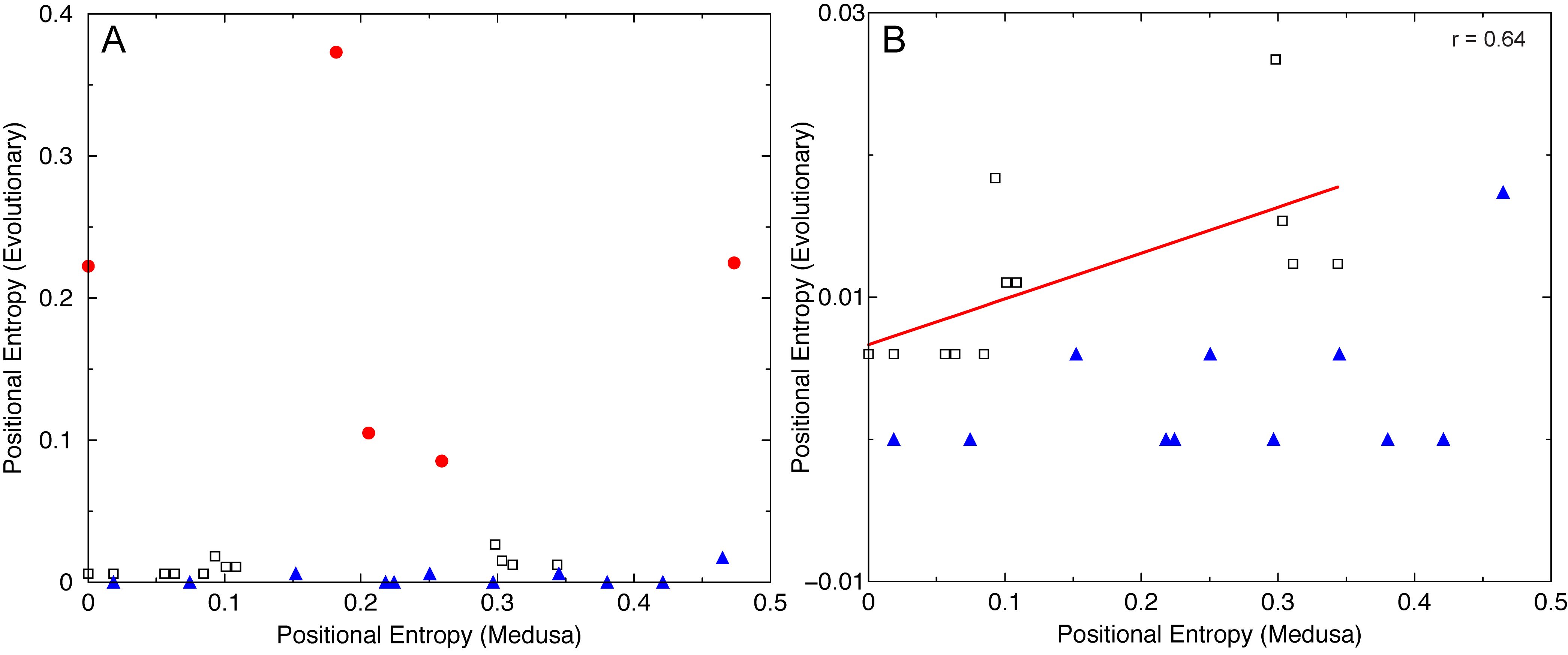

Supplement: Figure S3 — Distinct conservation profiles of three sets of residues in H3. Residues that feature much higher evolutionary entropy compared to other positions are shown as red circles (A). Residues featuring low evolutionary entropy, indicating conservation much higher than required by stability are indicated as blue triangles (A,B). Residues featuring evolutionary entropy that has modest correlation with Medusa-derived entropy are shown as black open squares (A,B). The red line indicates the linear regression (r = 0.64) between Medusa-derived entropy and evolutionary entropy for positions shown in black open squares (B). (0.49 MB TIF) [file pcbi.1001042.s003.tif]

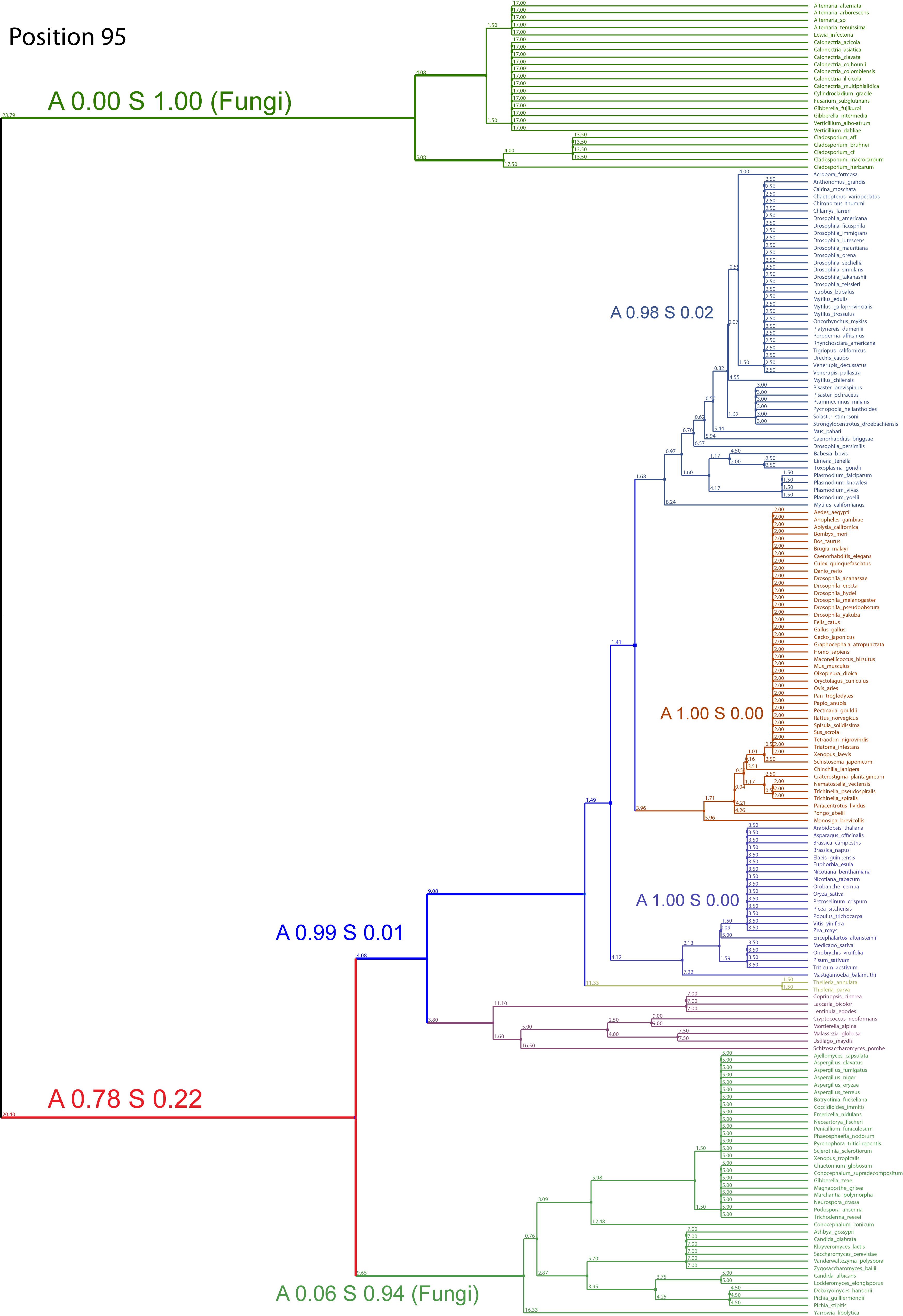

Supplement: Figure S4 — Tree-based conservation profile of position 95 in H3. The conservation of position 95 is determined at each node of the phylogenetic tree constructed from the multiple sequence alignment of H3. We observe that the nodes representing species mostly from kingdom Fungi, have a different preferred amino acid (Serine) compared to other nodes (Alanine), indicating tree-based inheritance. (1.52 MB TIF) [file pcbi.1001042.s004.tif]

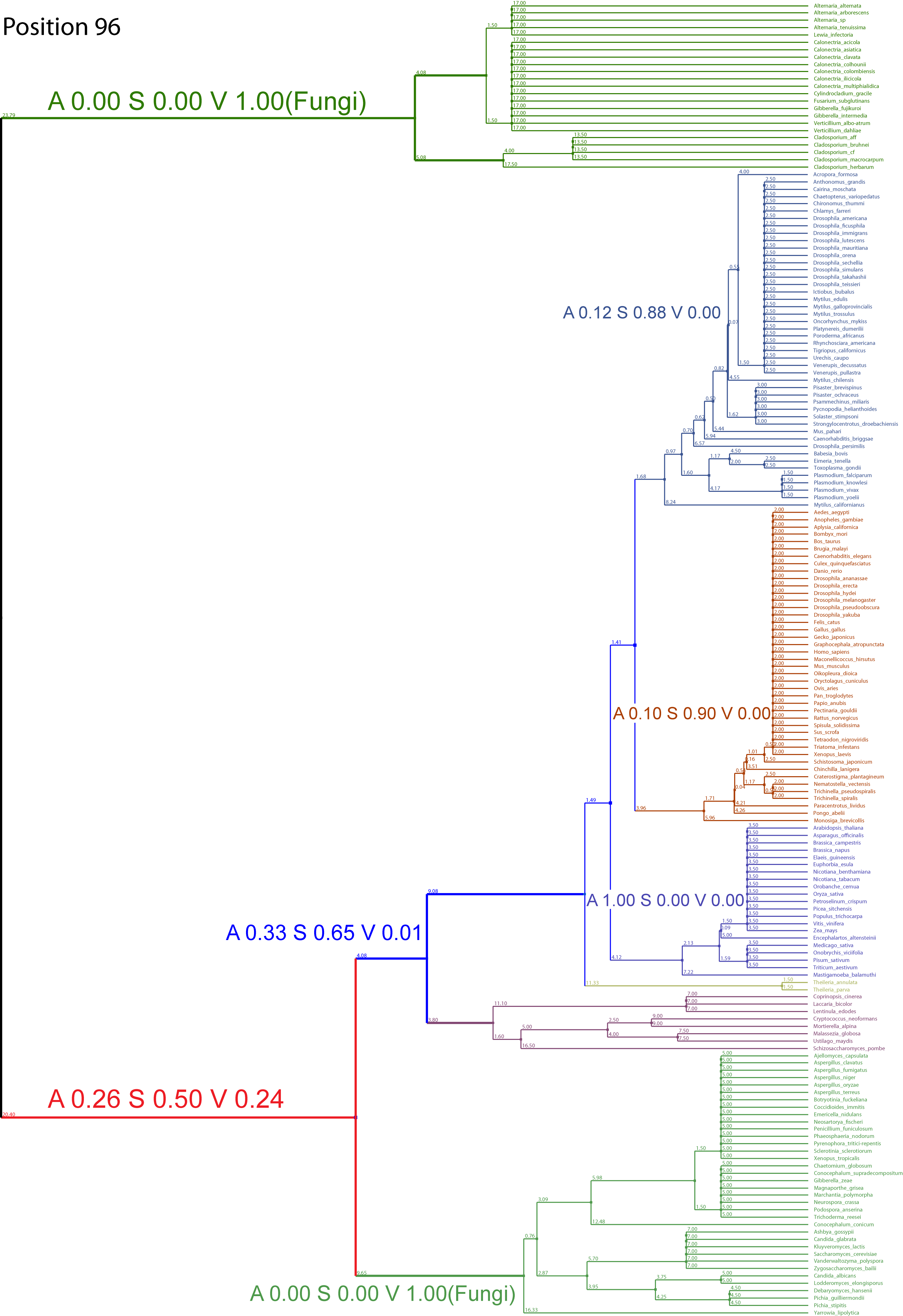

Supplement: Figure S5 — Tree-based conservation profile of position 96 in H3. The conservation of position 96 is determined at each node of the phylogenetic tree constructed from the multiple sequence alignment of H3. We observe that the nodes representing species mostly from kingdom Fungi, have a different preferred amino acid (Serine) compared to the node containing plant kingdom (Alanine), the node containing Homo sapiens and many species of genus Drosophila (Serine), indicating tree-based inheritance. (1.54 MB TIF) [file pcbi.1001042.s005.tif]

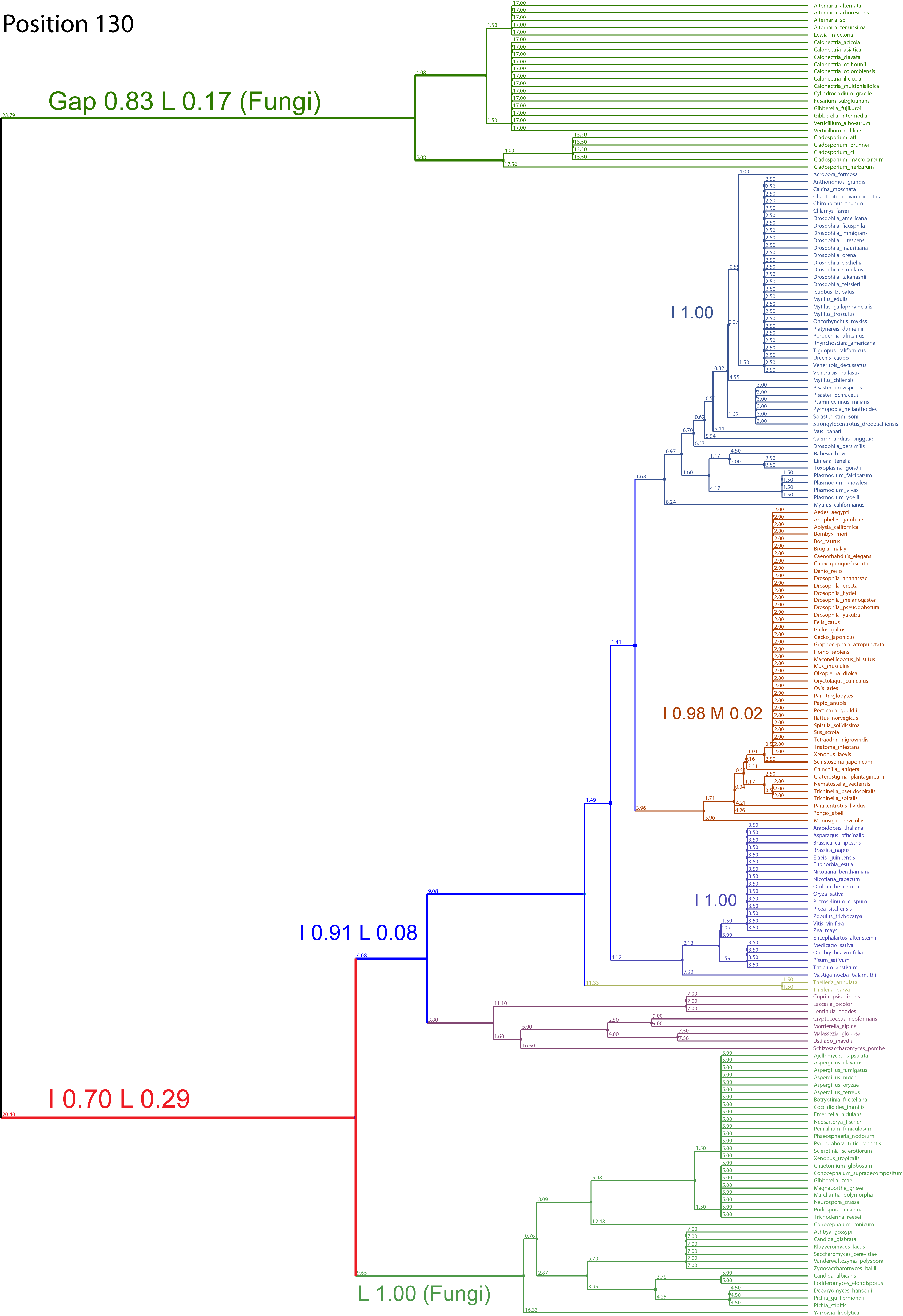

Supplement: Figure S6 — Tree-based conservation profile of position 130 in H3. The conservation of position 130 is determined at each node of the phylogenetic tree constructed from the multiple sequence alignment of H3. We observe that the nodes representing species mostly from kingdom Fungi, have a different preferred amino acid (Leucine) compared to other nodes (Isoleucine), indicating tree-based inheritance. (1.52 MB TIF) [file pcbi.1001042.s006.tif]

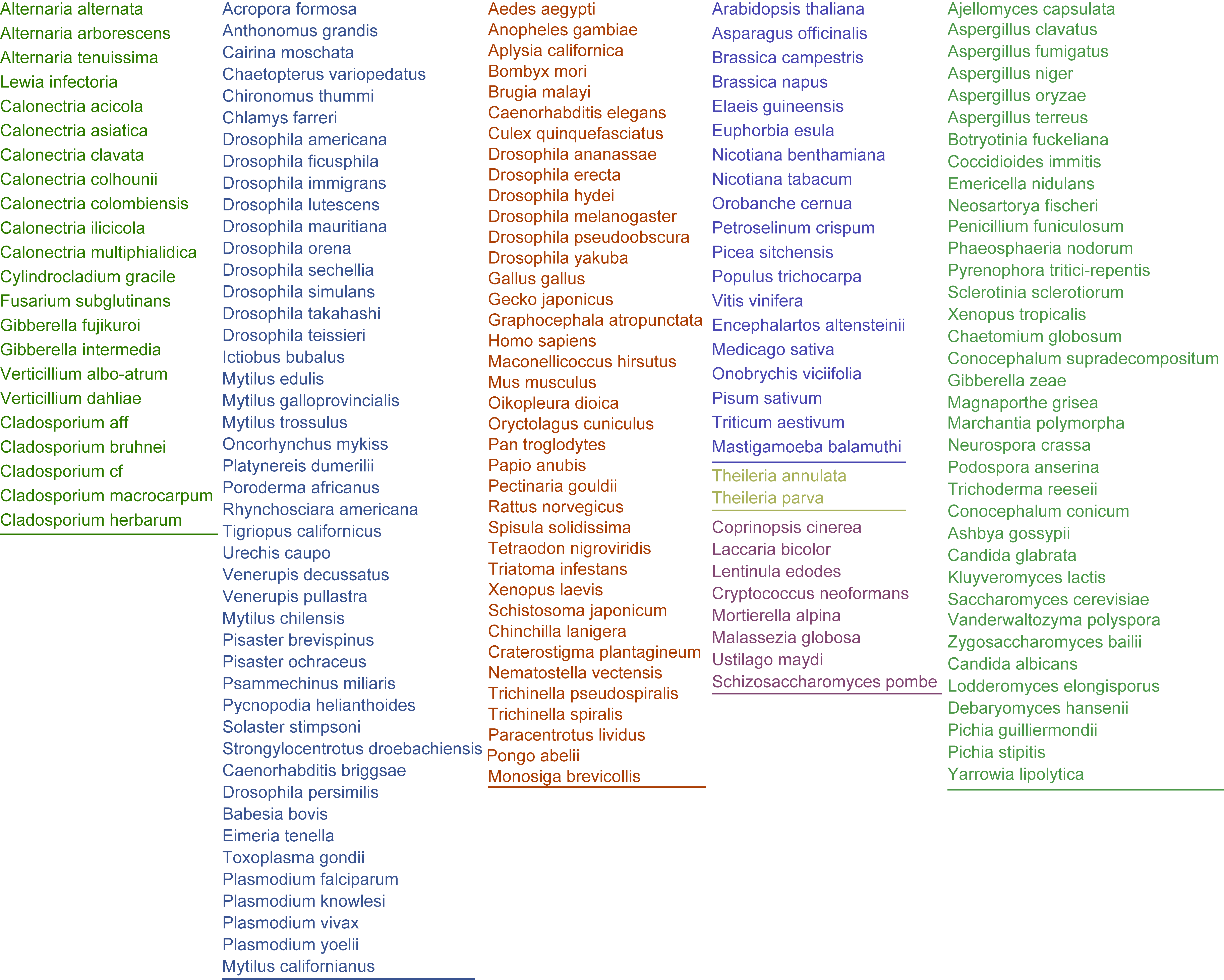

Supplement: Figure S7 — Organisms whose H3 sequences were used to construct the phylogenetic tree. The color coding of organisms is based on the nodes represented in the tree presented in Figures S3,S4 and S5. (2.83 MB TIF) [file pcbi.1001042.s007.tif]
