# Supplementary material for: Thermodynamic Stability of Histone H3 Is a Necessary but not Sufficient Driving Force for its Evolutionary Conservation
Source: PLoS Comput Biol. 2011 Jan 6;7(1):e1001042. doi: 10.1371/journal.pcbi.1001042 (PMC3017104; doi:10.1371/journal.pcbi.1001042)
Supplement: Table S1 — List of mutations in H3 extracted from the HistoneHits database. (0.05 MB DOC) [file pcbi.1001042.s008.doc]

Table S1. List of mutations in H3 extracted from the HistoneHits database.

| Mutation | Experimental Score^1^ | Medusa ∆∆G (kcal/mol) |
| --- | --- | --- |
| I51A | -2.0 | 4.4 |
| L61A | 0.0 | 2.0 |
| F67A | -0.7 | 6.3 |
| L70A | 0.0 | 5.5 |
| V71A | 0.0 | 3.6 |
| I74A* | -0.5 | 4.0 |
| A75S | 0.0 | -0.1 |
| A88S* | -0.5 | 0.5 |
| A91S* | -0.5 | 0.4 |
| L92A* | -1.0 | 4.6 |
| Q93A | -1.3 | 2.8 |
| Q93E | 0.0 | 2.0 |
| S95A | 0.0 | -1.3 |
| S95D | -2.0 | 1.5 |
| V96A | 0.0 | 1.0 |
| L100A | 0.0 | 5.5 |
| L103A | -1.3 | 3.7 |
| F104A* | -2.0 | 4.9 |
| T107A | 0.0 | 2.7 |
| T107D | -2.0 | 9.3 |
| A110S | -0.5 | 0.9 |
| A110S* | -0.5 | -0.4 |
| A111S* | -0.5 | 2.0 |
| H113A | -1.5 | 8.1 |
| H113Q | -2.0 | 3.9 |
| A114S | -0.5 | 0.1 |
| I119A | -1.3 | 2.8 |
| D123A | -1.3 | -0.4 |
| D123N | -2.0 | 0.3 |
| I124A* | -2.0 | 4.2 |
| L126A | -1.0 | 4.8 |
| A127S | -0.5 | 1.4 |
| L130A | -1.3 | 10.1 |
| R131A | -1.0 | 0.0 |
| R131K | -0.5 | 0.3 |

^1^Average of the 5-point phenotypic value from all the lethality assays of the mutant submitted to the database. A score of -0.67 was considered significantly affecting viability. *The results of temperature sensitivity assay are used for these mutants, as these mutants were lethal only at higher temperatures.
