# Supplementary material for: Thermodynamic Stability of Histone H3 Is a Necessary but not Sufficient Driving Force for its Evolutionary Conservation
Source: PLoS Comput Biol. 2011 Jan 6;7(1):e1001042. doi: 10.1371/journal.pcbi.1001042 (PMC3017104; doi:10.1371/journal.pcbi.1001042)
Supplement: Table S2 — List of mutations in H4 extracted from the HistoneHits database. (0.05 MB DOC) [file pcbi.1001042.s009.doc]

**Table S**2. List of mutations in H4 extracted from the HistoneHits database.

| Mutation | Experimental Score^1^ | Medusa ∆∆G (kcal/mol) |
| --- | --- | --- |
| I29A* | -1.0 | 3.6 |
| A33S | 0.0 | 0.3 |
| I34A | -1.3 | 5.8 |
| L37A* | -2.0 | 4.0 |
| A38S* | -1.0 | 0.7 |
| V43A | 0.0 | 4.3 |
| I50A | 0.0 | 4.4 |
| V54A | 0.0 | 2.4 |
| L58A* | -0.5 | 4.6 |
| L62A* | -1.0 | 5.6 |
| V65A | 0.0 | 3.0 |
| I66A* | -1.0 | 3.8 |
| S69A | 0.0 | -2.0 |
| S69D | -2.0 | 4.2 |
| T73A | 0.0 | 0.5 |
| T73D | -2.0 | 3.6 |
| V81A* | -0.5 | 2.4 |
| V86A* | -0.5 | 2.5 |
| A89S | 0.0 | 3.8 |
| L90A | -2.0 | 4.0 |
| T96A | 0.0 | -0.5 |
| T96D | 0.0 | 1.3 |
| L97A | 0.0 | 2.4 |
| Y98A | -0.5 | 7.5 |
| Y98E | -1.0 | 10.5 |
| Y98F | 0.0 | -2.8 |
| G99A* | -0.5 | 4.7 |

^1^Average of the 5-point phenotypic value from all the lethality assays of the mutant submitted to the database. A score of -0.67 was considered significantly affecting viability. *The results of temperature sensitivity assay are used for these mutants, as these mutants were lethal only at higher temperatures.
