# Supplementary material for: Thermodynamic Stability of Histone H3 Is a Necessary but not Sufficient Driving Force for its Evolutionary Conservation
Source: PLoS Comput Biol. 2011 Jan 6;7(1):e1001042. doi: 10.1371/journal.pcbi.1001042 (PMC3017104; doi:10.1371/journal.pcbi.1001042)
Supplement: Table S3 — Evolutionary and Medusa positional entropy values of buried and interface residues of H3. (0.05 MB DOC) [file pcbi.1001042.s010.doc]

**Table S**3. Evolutionary and Medusa positional entropy values of buried and interface residues of H3.

| Residue | Evolutionary Entropy^1^ | Medusa Entropy^2^ |
| --- | --- | --- |
| I51 | 0.00 | 0.30 |
| L61 | 0.00 | 0.42 |
| F67 | 0.01 | 0.25 |
| L70 | 0.01 | 0.11 |
| V71 | 0.01 | 0.31 |
| I74 | 0.02 | 0.30 |
| A75 | 0.00 | 0.38 |
| A88 | 0.00 | 0.22 |
| A91 | 0.00 | 0.22 |
| L92 | 0.00 | 0.07 |
| Q93 | 0.01 | 0.34 |
| S95 | 0.22 | 0.47 |
| V96 | 0.37 | 0.18 |
| L100 | 0.01 | 0.15 |
| L103 | 0.02 | 0.09 |
| F104 | 0.01 | 0.02 |
| T107 | 0.01 | 0.34 |
| A110 | 0.11 | 0.21 |
| A111 | 0.01 | 0.06 |
| H113 | 0.01 | 0.00 |
| A114 | 0.02 | 0.46 |
| I119 | 0.01 | 0.08 |
| D123 | 0.03 | 0.30 |
| I124 | 0.09 | 0.26 |
| L126 | 0.01 | 0.06 |
| A127 | 0.01 | 0.10 |
| L130 | 0.22 | 0.00 |
| R131 | 0.00 | 0.02 |

^1^Entropy values obtained from HSSP database have been normalized by ln(20), the maximal possible entropy, so that the range of entropy values is between 0-1. ^2^Normalized entropy obtained using the residue propensities in Medusa calculations as described in the Methods.
