# Supplementary material for: Thermodynamic Stability of Histone H3 Is a Necessary but not Sufficient Driving Force for its Evolutionary Conservation
Source: PLoS Comput Biol. 2011 Jan 6;7(1):e1001042. doi: 10.1371/journal.pcbi.1001042 (PMC3017104; doi:10.1371/journal.pcbi.1001042)
Supplement: Table S4 — Evolutionary and Medusa positional entropy values of buried and interface residues of H4. (0.04 MB DOC) [file pcbi.1001042.s011.doc]

Table S4. Evolutionary and Medusa positional entropy values of buried and interface residues of H4.

| Residue | Evolutionary Entropy^1^ | Medusa Entropy^2^ |
| --- | --- | --- |
| I29 | 0.08 | 0.44 |
| A33 | 0.16 | 0.51 |
| I34 | 0.09 | 0.04 |
| L37 | 0.08 | 0.25 |
| A38 | 0.02 | 0.19 |
| V43 | 0.01 | 0.04 |
| I50 | 0.15 | 0.15 |
| V54 | 0.26 | 0.45 |
| L58 | 0.06 | 0.16 |
| L62 | 0.09 | 0.12 |
| V65 | 0.12 | 0.29 |
| I66 | 0.17 | 0.31 |
| S69 | 0.22 | 0.34 |
| T73 | 0.09 | 0.36 |
| V81 | 0.03 | 0.24 |
| V86 | 0.05 | 0.19 |
| A89 | 0.09 | 0.08 |
| L90 | 0.02 | 0.03 |
| T96 | 0.18 | 0.39 |
| L97 | 0.07 | 0.08 |
| Y98 | 0.01 | 0.02 |
| G99 | 0.02 | 0.00 |

^1^Entropy values obtained from HSSP database have been normalized by ln(20), the maximal possible entropy, so that the range of entropy values is between 0-1. ^2^Normalized entropy obtained using the residue propensities in Medusa calculations as described in the Methods.
