# Supplementary material for: Thermodynamic Stability of Histone H3 Is a Necessary but not Sufficient Driving Force for its Evolutionary Conservation
Source: PLoS Comput Biol. 2011 Jan 6;7(1):e1001042. doi: 10.1371/journal.pcbi.1001042 (PMC3017104; doi:10.1371/journal.pcbi.1001042)
Supplement: Table S5 — List of residues in H3 that feature high conservation. 1 Entropy values obtained from HSSP database have been normalized by ln(20), the maximal possible entropy, so that the range of entropy values is between 0–1. 2 Normalized entropy obtained using the residue propensities in Medusa calculations as described in the Methods. (0.04 MB DOC) [file pcbi.1001042.s012.doc]

**Table S5. List of residues in H3 that feature high conservation**.

| Residue | Evolutionary Entropy^1^ | Medusa Entropy^2^ | Buried /Interface |
| --- | --- | --- | --- |
| I51 | 0.00 | 0.30 | Buried |
| L61 | 0.00 | 0.42 | Buried |
| F67 | 0.01 | 0.25 | Buried |
| A75 | 0.00 | 0.38 | Buried |
| A88 | 0.00 | 0.22 | Buried |
| A91 | 0.00 | 0.22 | Buried |
| L92 | 0.00 | 0.07 | Buried |
| Q93 | 0.01 | 0.34 | Buried |
| L100 | 0.01 | 0.15 | Buried |
| A114 | 0.02 | 0.46 | Interface |
| R131 | 0.00 | 0.02 | Interface |

^1^Entropy values obtained from HSSP database have been normalized by ln(20), the maximal possible entropy, so that the range of entropy values is between 0-1. ^2^Normalized entropy obtained using the residue propensities in Medusa calculations as described in the Methods.
