# Supplementary material for: Thermodynamic Stability of Histone H3 Is a Necessary but not Sufficient Driving Force for its Evolutionary Conservation
Source: PLoS Comput Biol. 2011 Jan 6;7(1):e1001042. doi: 10.1371/journal.pcbi.1001042 (PMC3017104; doi:10.1371/journal.pcbi.1001042)
Supplement: Table S6 — Yeast strains used in the study. (0.04 MB DOC) [file pcbi.1001042.s013.doc]

Table S6. Yeast strains used in the study.

| Strain | Genotype | References |
| --- | --- | --- |
| WZY42 | *MAT*a, *ura3–52*, *lys2–801*, *ade2–101*, *trp1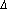63*, *his3200*, *leu21*, *hht1-hhf1*::pWZ405-F2F9-LEU2,*hht2-hhf2*::pWZ403-F4F10-HIS3, Ycp50-copyII (*HHT2-HHF2*) | Zhang et al. 1998^1^ |
| WZY42 H3-H113A | Isogenic to WZY42, plus Ycp50-copyII *(HHT2(*H113A) *-HHF2)* | This study |
| WZY42 H3-L126A | Isogenic to WZY42, plus Ycp50-copyII *(HHT2(*L126A) *-HHF2)* | This study |
| WZY42 H3-L130A | Isogenic to WZY42, plus Ycp50-copyII *(HHT2(*L130A) *-HHF2)* | This study |
| YBL574 | MATa, leu2Δ1, his3Δ200, ura3-52, trp1Δ63, lys2-128δ, (hht1-hhf1)ΔLEU2(hht2-hhf2)Δ::HIS3 Ty912Δ35-lacZ::his4, {pDM9-HHT1-HHF1-URA3} | Carrozza et al. 2005² |
| YBL574 H3-H113A | Isogenic to YBL574, plus Ycp50-copyII *(HHT2(*H113A) *-HHF2)* | This study |
| YBL574 H3-L126A | Isogenic to YBL574, plus Ycp50-copyII *(HHT2(*L126A) *-HHF2)* | This study |
| YBL574 H3-L130A | Isogenic to YBL574, plus Ycp50-copyII *(HHT2(*L130A) *-HHF2)* | This study |

^1^Zhang, W; Bone, JR; Edmondson, DG; Turner, BM; Roth, SY. Essential and redundant functions of histone acetylation revealed by mutation of target lysines and loss of the Gcn5p acetyltransferase. *EMBO J.* 1998;17:3155–3167.

^2^M.J. Carrozza, B. Li, L. Florens, T. Suganuma, S.K. Swanson, K.K. Lee, W.J. Shia, S. Anderson, J. Yates, M.P. Washburn and J.L. Workman, Histone H3 methylation by Set2 directs deacetylation of coding regions by Rpd3S to suppress spurious intragenic transcription**,** *Cell* **123** (2005), 581–592.
